# Use of Human Neurons Derived via Cellular Reprogramming Methods to Study Host-Parasite Interactions of *Toxoplasma gondii* in Neurons

**DOI:** 10.3390/cells6040032

**Published:** 2017-09-23

**Authors:** Sandra K. Halonen

**Affiliations:** Department of Microbiology and Immunology, Montana State University, Bozeman, MT 59718, USA; shalonen@montana.edu; Tel.: +406-994-5351

**Keywords:** cysts, bradyzoites, chronic Toxoplasmosis, Cerebral Toxoplasmosis, latent infection, spontaneous cyst formation, *T. gondii* in neurons

## Abstract

*Toxoplasma gondii* is an intracellular protozoan parasite, with approximately one-third of the worlds’ population chronically infected. In chronically infected individuals, the parasite resides in tissue cysts in neurons in the brain. The chronic infection in immunocompetant individuals has traditionally been considered to be asymptomatic, but increasing evidence indicates that chronic infection is associated with diverse neurological disorders such as schizophrenia, cryptogenic epilepsy, and Parkinson’s Disease. The mechanisms by which the parasite exerts affects on behavior and other neuronal functions are not understood. Human neurons derived from cellular reprogramming methods offer the opportunity to develop better human neuronal models to study *T. gondii* in neurons. Results from two studies using human neurons derived via cellular reprogramming methods indicate these human neuronal models provide better in vitro models to study the effects of *T. gondii* on neurons and neurological functions. In this review, an overview of the current neural reprogramming methods will be given, followed by a summary of the studies using human induced pluripotent stem cell (hiPSC)-derived neurons and induced neurons (iNs) to study *T. gondii* in neurons. The potential of these neural reprogramming methods for further study of the host-parasite interactions of *T. gondii* in neurons will be discussed.

## 1. Introduction

*Toxoplasma gondii* is an obligate intracellular protozoan parasite that is ubiquitous in nature, with estimates that approximately one-third of the worlds’ population is chronically infected [1]. In chronically infected individuals, *T. gondii* resides primarily in tissue cysts located within neurons in the brain, which are maintained for the lifetime of the individual [2,3]. Chronic *Toxoplasma* infection in immunocompetent individuals has typically been considered asymptomatic, but there is increasing evidence for association of the chronic infection with neuropsychiatric disorders such as schizophrenia and suicidal behavior, neurological disorders such as cryptogenic epilepsy, and a possible association with neurodegenerative disorders such as Parkinson’s Disease and Alzheimer’s Disease [4,5,6,7,8,9,10,11,12,13,14,15,16,17,18,19,20,21,22,23,24,25,26]. The mechanisms by which the parasite exerts these diverse effects on behavioral and neurological functions are not understood.

Studies in chronically infected mice indicate *T. gondii* exerts direct effects on neuronal functions that could contribute to behavioral and neurological effects on the host. For example the parasite increases levels of dopamine, induces changes that influence neuronal connectivity, including defects in functionality of synaptic neurotransmission, and there is some indication that infected neurons are functionally silenced [27,28,29]. Additionally, *Toxoplasma* injects host cells proteins into the neuron host cells that it infects, as well as neighboring uninfected neurons suggesting the parasite could affect a field of neurons surrounding the infected neuron [30]. All of above suggest that there are mechanism(s) by which the parasite could affect neurotransmission and other neurological functions of the host. However, neither the parasite effects on neurons nor the underlying mechanisms by which the parasite affects neurons are well understood.

A better understanding of the cellular and molecular basis of the parasites effects on neurons is limited by lack of suitable in vitro culture systems to study the parasite in neurons. Previous in vitro models of *Toxoplasma*-infected neurons have consisted of study in human neuronal cell lines [31,32]. However while neuronal cell lines can be induced to express neuronal characteristics such as expression of dendritic processes and production of synapses, they are often derived from neuronal tumors, and thus better human neuronal in vitro models are still needed. Primary neuronal cultures derived from rodents (mice or rats) have also been used to study *T. gondii*, however these primary neuronal cultures typically contain astrocytes, which preferentially support replication of tachyzoites, the rapidly replicating form of the parasite, which overgrows the culture and prohibits development of the cysts in neurons, the predominant form present in the chronic infection [33,34,35]. Primary human neuronal cultures have also been used to study *T. gondii*, but similar to rodent primary neuronal cultures, astrocytes preferentially support tachyzoites, which prohibits study of cyst development in neurons [36]. Additionally human neurons are hard to obtain and thus the numbers of cells available for study are limited. Better human neuronal models are needed to study the effects of the parasite in neurons and to further our understanding of the mechanism(s) by which the parasite affects neuronal functions.

Human neurons derived from somatic cells via cellular reprogramming methods such as human induced pluripotent stem cells (hiPSCs) or neurons derived directly from somatic cells (induced neurons or iNs), offer the opportunity to develop human neuronal models that overcome many of the limitations of the current in vitro models. Cellular reprogramming technologies can produce relatively pure populations of neurons (up to 90% or greater) that have functional neuronal characteristics such as the expression of dendritic processes and axons, and that respond to synaptic stimulation [37]. Neurons derived from cellular reprogramming methods have been investigated extensively for use in the study of neurobiological diseases, and hence many methods for differentiation and cultivation of reprogrammed human neurons have been established and are relatively easy to use [37,38,39,40,41,42]. As such, reprogrammed human neurons are now more accessible for use in a broader research context. The application of stem cell-derived cultures to study protozoan parasites for which there are often no good in vitro systems, has begun to be used in the study of a few protozoan parasites [43]. For *T. gondii*, an obligate intracellular parasite that infects neurons in the brain, use of reprogrammed human neurons affords exciting new opportunities to develop in vitro human neuronal models to explore the host-parasite relationships of *T. gondii* in neurons.

In this review, the current cellular reprogramming methods used to derive human neurons will first be reviewed, with a brief discussion of existing limitations of cellular reprogramming technology, specifically as it relates to uses in disease modeling. Then, the use of reprogrammed human neurons as a model in vitro system to study the biology of *T. gondii* in neurons and to address outstanding questions about Cerebral Toxoplasmosis and host-parasite relationships in neurons in the brain, will be discussed.

## 2. Generation of iPSCs and Development of the Field of Cellular Reprogramming

The development of iPSC technology originated from the studies by Takahashi and Yamanaka in 2006 and 2007, who reported that the ectopic expression of four transcription factors, *Oct 3/4*, *Sox2*, *Klf4*, and *c-Myc* (OSKM) could reprogram somatic cells into pluripotent cells that were similar to embryonic stem cells, being self-renewing and able to be directed to differentiate into a variety of differentiated cell types [44,45]. This area of research has experienced tremendous growth in the 10 years since Takahashi and Yamanaka’s discovery, with different methods developed to convert a somatic cell into another differentiated cell type. This field is now more broadly called cellular reprogramming. Numerous improvements to cellular reprogramming strategies have been developed, involving development of non-integrative methods and other techniques to limit potential for tumorogenesis and to increase efficiency. The advances in reprogramming methods are the subject of several recent, comprehensive reviews to which the reader is referred for further details on this subject [37,38,39,46,47].

The advent of cellular reprogramming technology has been especially useful for the study of neurobiological disorders, where research has been complicated by lack of access of neuronal tissues and the complex nature of many neurological disorders and where animal models do not necessarily recapitulate the human phenotype of neurological disorders due to inherent structural, developmental and behavioral differences between mice and human nervous systems [37,40,48,49,50,51]. Use of these cellular reprogramming-based neuronal models to address pathogenesis, disease mechanisms and drug screening has lead to the idea of ‘disease in a dish models’ of neurological disorders. Neurological disease models using reprogrammed human neurons have been successfully developed for neuropsychiatric diseases such as schizophrenia, neurodevelopmental disorders such as Alzheimer's disease, some motor neuron disorders such as amyotrophic lateral sclerosis (ALS), and several other neurological disorders [39,41,42,48,49,50,52].

### 2.1. Differentiation Protocols to Derive Neurons and Neuronal Subtypes

**Figure 1:**
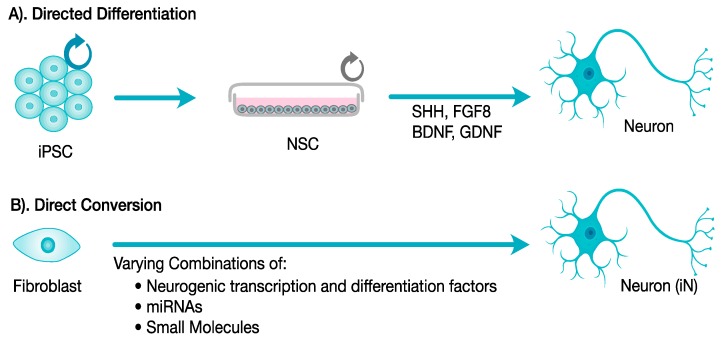
Directed differentiation vs. direct conversion methods to derive neurons. In Directed Differentiation (**A**) an iPSC is first created and then differentiated into a neural stem cell (NSC) that is self-renewing and can be induced to differentiate into neurons via the transcription and growth factors such as sonic hedgehog (SHH), fibroblast growth factor 8 (FGF8), which induce differentiate into dopaminergic neurons, brain-derived neurotrophic factor (BDNF), and glial-derived neurotrophic factor (GDNF) to promote neuronal maturation. In Direct Conversion (**B**) a somatic cell, such as a fibroblast, is directly converted into a neuron via a combination of neurogenic transcription and differentiation factors, miRNAs and small molecules, producing neurons called induced neurons (iNs).

Two basic methods of deriving neurons from somatic cells have been established, directed differentiation and direct conversion [46]. Directed differentiation involves generation of iPSCs and subsequent derivation into neural stem cells (NSCs), which can then be differentiated into neurons via transcription and growth factors (Figure 1A). Direct conversion converts a somatic cell, such as a fibroblast, directly into a neuron without going through an intermediate pluripotent stage generating iNs (Figure 1B). Both methods can generate neurons that express neuronal markers, neuronal morphological characteristics such as dendrites and axons, and have been shown to be functionally active and able to respond to synaptic stimulation [46,47]. Directed differentiation has the advantage that the hiPSC-derived NSCs can generate a broad range of neuronal subtypes, such as glutamatergic, dopaminergic, serotonergic or GABAergic neurons, using different transcription factors [53,54]. For example, the transcription factors Sonic Hedgehog (SHH) and FGF8 direct NSCs toward dopaminergic neurons, while FGF2, SHH, retinoic acid, and activin direct NSCs toward motor neurons, and cortical neurons expressing the neurotransmitter GABA can develop in the absence of specific transcription factors [54]. In addition, hiPSC-derived NSCs can be induced to differentiate into other neural cells such as astrocytes and oligodendrocytes via addition of other transcription factors. Direct conversion methods, also called lineage conversion or transdifferentiation, can generate neurons more rapidly than directed differentiation (~14 days) and has the potential to produce more pure populations of neurons (up to 90% or greater), thus overcoming some of limitations of directed differentiation [47]. The rapid derivation of neurons via direct conversion is advantageous for use of patient-specific cells for production of ‘disease in a dish’ neuronal models. A variety of neuronal subtypes, including dopaminergic, glutamatergic, and GABAergic neurons have now been differentiated using direct conversion, although not all neuronal subtypes have been successfully differentiated [41].

### 2.2. Neural Cellular Reprogramming: Existing Limitations and Implications to Disease Modeling

While many advances in cellular reprogramming technologies and neurological differentiation protocols have been developed, there are still several significant limitations for these methods. First, directed differentiation processes often require long periods of time (months) to generate mature neurons that often leads to heterogeneity within cultures [46]. Secondly, protocols that generate a specific neuronal subtype often produce neuronal subtypes with varying frequency. Additionally, a major limitation using directed differentiation is that many hiPSC-derived neurons retain immature characteristics and do not reach full maturation [37,55,56,57]. For direct conversion the efficiency of differentiation of the target cell population varies widely with efficiencies ranging from <10% to >90%, amongst different differentiation protocols [58]. Despite these limitations, improvements in both directed differentiation and direct conversion protocols continue to be developed to improve the homogeneity of NSC cell lines generated from iPSCs, methods for enrichment for NSC subtypes, and improvements in the neural reprogramming efficiency of direct conversion methods via the use of small molecules [47,59,60,61,62,63,64].

In addition to the above limitations, it is now clear that a large amount of genomic instability and epigenetic aberrations exists in hiPSC cell lines and iNs [65]. The reprogramming process itself is now recognized to involve an epigenetic remodeling process consisting of genome-wide DNA methylation and histone modifications (acetylation and methylation) [65]. A large number of the alterations to the epigenome arises from the reprogramming process itself, with OKSMs inducing changes in pluripotency genes that erase epigenetic signatures and result in the cell adopting the epigenome of a stem cell [46,65,66]. However, genome-wide genetic and epigenetic analyses have revealed there is a persistence of epigenetic memory in many PSCs, and that different DNA methylation signatures and differing degrees of genetic aberrations exist amongst different iPSCs [67]. These subtle differences in pluripotent stem cells raise the question of whether iPSCs truly recapitulate certain diseases and introduce variability that compromises disease modeling accuracy [68]. Somatic cell reprogramming and the epigenetic mechanisms underlying reprogramming are not yet fully understood, but clearly a better understanding of these processes is necessary to improve clinical safety of iPSCs and for accuracy of disease modeling. For further information on the details of epigenetic events that can arise during cellular reprogramming, the underlying mechanisms and specifically the implications for neurological disease modeling, the reader is referred to several recent reviews on these topics [65,66,69].

## 3. Use of Reprogrammed Human Neurons to Study Cerebral Toxoplasmosis

**Figure 2:**
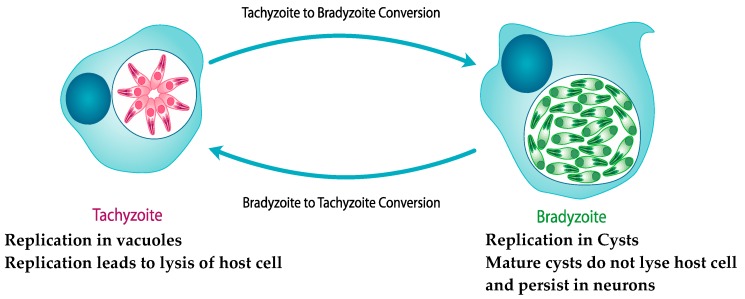
Parasite stages of *T. gondii* in host cells in the brain. The rapidly replicating tachyzoite stage replicates in vacuoles leading to lysis of the host cell usually within 36–72 h after infection of the host cell, and the slowly replicating bradyzoite stage replicates in cysts that do not lyse host cells and persist in neurons for the lifetime of the host.

*T. gondii* consists of two stages in the brain, the rapidly replicating tachyzoite stage, which replicates in parasitophorous vacuoles, and the slowly replicating bradyzoite stage, would replicates in cysts (Figure 2). Tachyzoites enter the brain shortly after infection and initially replicate in neurons as well as astrocytes and microglia, but convert to the bradyzoite stage, producing cysts located in neurons. Intraneural cysts last for the lifetime of the host, with periodic cyst rupture and bradyzoite to tachyzoite conversion thought to occur during the chronic infection [70,71,72]. However, the host immune response via IFN-γ mediated mechanisms controls the replication of tachyzoites in the brain [73,74,75]. The host immune response is not able to clear the intraneural cysts. A good in vitro human neuronal model ideally would support replication of both tachyzoite and bradyzoite stages and would allow the development and maturation of cysts, the most relevant stage of the parasite in the chronic infection and the least well understood aspect of the biology of the parasite in the brain.

**Figure 3:**
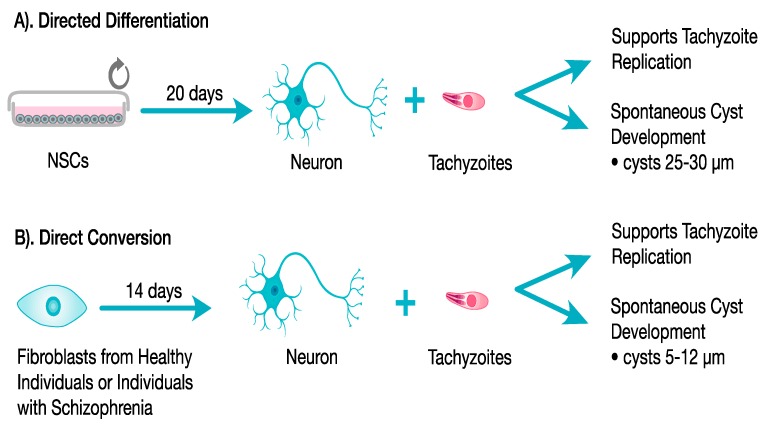
Studies of *T. gondii* using reprogrammed human neurons. Using a directed differentiation method (**A**), NSCs were used to derived human neurons in 20 days and then infected with *T. gondii* tachyzoites. Using a Direct Conversion method (**B**), starting from fibroblasts derived either from healthy controls or individuals with Schizophrenia, induced human neurons (iNs) were generated in 14 days and then infected with *T. gondii* tachyzoites. In both human neuronal models, neurons supported tachyzoite replication and bradyzoite replications, which lead to spontaneous cyst development. In fibroblasts for example, typically less than 10% tachyzoite vacuoles spontaneously convert to cysts.

To date, two studies have investigated the use of reprogrammed human neurons to study *T. gondii* in neurons [76,77]. One study used a directed differentiation method, starting from a hiPSC-derived NSC population to derive human neurons (Figure 3A), and the other study used a direct conversion method, starting from fibroblasts from normal individuals and schizophrenia patients to generate iNs (Figure 3B). Neurons derived from both methods could be efficiently infected with *T. gondii* and supported replication of the tachyzoite stage. Additionally, both human neuronal models supported bradyzoite replication and cyst development, the dominant forms found in the chronic infection (Figure 3). Interestingly both neuronal methods supported spontaneous cyst formation, which does not occur in most non-neuronal cells and indicates that the neuron host cell environment itself influences stage conversion, an aspect of the host-parasite relationship in the brain that previously was not known. Additionally, the directed differentiation method using hiPSC-derived neurons supported a large percentage of vacuoles converting to cysts (~90%) with many attaining sizes (20–30 μm in diameter) and morphological characteristics of mature cysts (unpubl.; S, Halonen), indicating that this method may be an ideal human neuronal model system to use for bradyzoite growth and cyst development studies as well as anti-bradyzoite/cyst drug studies.

In summary, these studies using human neurons derived via cellular reprogramming techniques indicate that both of these methods provide good in vitro human neuronal models to study the tachyzoite stage, bradyzoite stage, and cyst development in human neurons.

### 3.1. Use of Reprogrammed Human Neuronal Models to Study the Host/Parasite Relationships in Neurons and to Address Outstanding Questions about Cerebral Toxoplasmosis

The basic biology of *T. gondii* in the brain is relatively well established, derived primarily from studies of mice chronically infected with *Toxoplasma* and in vitro culture systems. However there is much about the host-parasite relationship in neurons and questions about the biology of the parasite in the brain that are not understood or easily addressed using in vivo murine models of chronic toxoplasmosis, or existing in vitro cell culture systems, which typically consists of the study of *Toxoplasma* in non-neuronal host cells such as fibroblasts. For example, use of non-neural in vitro culture systems has revealed many mechanisms by which tachyzoites and to a lesser extent, bradyzoites, manipulate host cells including the modulation of cell signaling pathways, host cell cycle, apoptosis, and modulation of host cell transcription [78,79,80,81,82,83]. However, it is not known if tachyzoites and bradyzoites have similar effects in the neuron host cell. Additionally, as previously mentioned, studies in chronically infected mice indicate that the parasite has direct effects on neuronal functions such as affecting neuronal transmission and functionally silencing neurons, but little is understood about these effects in neurons or the underling mechanisms by which the parasite exerts these neuronal effects. Finally, a major gap in our knowledge is an understanding of bradyzoite biology and cysts, the dominant stages present in the chronic infection. Non-neural host cells have been effectively used to study tachyzoite-bradyzoite stage conversion and have allowed the discovery of mechanisms involved in bradyzoite differentiation [84,85,86,87,88]. However, these methods typically do not permit cyst development beyond a few days in culture, and hence our understanding of the biology of mature cysts and bradyzoite growth and development is lacking, and a better in vitro model to study these aspects of the parasites biology in the brain is of high importance. Finally, while it is well understood the immune response mediated primarily by the cytokine IFN-γ, is essential to control the chronic infection, the exact effects of IFN-γ or other immune components on parasite stage conversion in neurons, cyst maturation or cyst rupture, are not known.

The use of neurons derived via cellular reprogramming techniques overcome many of these limitations of existing in vivo and in vitro models. Additionally, results from these initial studies using reprogrammed human neurons indicate that they provide better in vitro human neuronal models to address outstanding questions regarding the host/parasite relationships in neurons and about the biology of the chronic phase of infection in the brain.

### 3.2. Advantages of Human Neuronal Models Using Reprogrammed Neurons and Applications to the Study of Cerebral Toxoplasmosis

Advantages of the use of human neurons derived via cellular reprogramming methods to the study of host-parasite interactions in neurons are outlined below, with the potential applications to the study of Cerebral Toxoplasmosis, as summarized in Table 1.

#### 3.2.1. Generation of Relatively Unlimited Supply of Pure, Mature Human Neurons

Reprogrammed neurons create a relatively unlimited supply of human neurons, which traditionally have been difficult to obtain and typically available only via access to fetal or autopsy tissues. The generation of relatively unlimited human neurons of high purity (up to 90% or more) would allow the cellular and molecular basis of effects of tachyzoite and bradyzoite stages in human neurons to be addressed in the context of a human neuron host cell, an advantage over existing in vitro rodent neuronal models. Transcriptional profiling of both tachyzoite and bradyzoite stages in human neurons for example could be done to help illuminate underlying molecular and cellular basis of the host/parasite relationship. Additionally, other questions such as the effects of IFN-γ on tachyzoite replication and stage conversion in neurons, the effect of the parasite on neuronal apoptosis, etc., could be addressed using either of these human neuronal models.

The use of hiPSC-derived neurons may be better for these purposes, as this method facilitates successive neuronal differentiations via generation of NSCs, which are self-renewing, and thus are amenable to high throughput experimentation. Several NSC cell lines have been created, which are available via NIH or commercially available from several sources, which also facilitates the ease of use of this method to generate human neurons. However it should be noted that epigenetic variability and genomic instabilities present in both iPSCs and iNs could affect neuronal functions, and hence interpretations of parasite/neuron interactions using reprogrammed human neurons need to be taken with some caution. It is also possible that the factors used to differentiate and/or maintain the neuronal differentiated state (i.e., BDNF and GNDF) may affect the parasite. Additional experiments to verify proposed mechanisms could be done with multiple iPSC/NSC cell lines, or with iNs from varying individuals, or experimental results could be verified in primary neurons or with in vivo studies, for example, to address these concerns.

#### 3.2.2. Generation of Human Neurons with Functional Characteristics

Reprogrammed human neurons have the functional characteristics of mature neurons, which is an advantage over many existing neural cell lines. Human neurons derived from hiPSCs and iNs both create human neurons that express phenotypic neuronal characteristics such as dendritic processes, the ability to generate action potentials and formation of synapses when cultured in the presence of astrocytes [53,54]. Thus functional aspects of the host-parasite interactions such as the effect of infection on neurotransmission, the synapse, or investigations into mechanisms by which infected neurons may become functionally silenced, as reports from in vivo studies suggest, could be addressed with these models [27,28].

#### 3.2.3. Disease-Specific Neurons Can Be Created

Both directed differentiation and direct conversion afford the possibility of deriving neurons from patients with Schizophrenia and other relevant neuronal disorders to be created. Use of patient-specific neurons would allow the effects of *T. gondii* infection to be studied in a disease-specific context. Additionally, the use of neurons derived from patients with different genetic mutations allows the assessment of specific genetic factors on neuronal susceptibility to *T. gondii* to be assessed. For example in the recent study in which schizophrenia patient iNs (SZ-iNs) were infected, it was found that while SZ-iNs with 22q11.2 deletion were infected at the same rate as iNs from non-SZ individuals (controls), the number of tachyzoites per infected iNs was higher in SZ-iNs with the 22q11.2 deletion as compared to the control iNs or SZ-iNs with another genetic deletion and also that cysts in either of SZ-iNs were smaller than control iNs [77]. Thus, the results from this study indicate some parasite/neuron interactions may be differentially affected in schizophrenia neurons, and illustrate that the potentially significant aspects of neuronal host cell/parasite interactions can be revealed using patient-specific neurons. iNs can be created relatively quickly, and may be more advantageous to use in generation of patient specific neurons than the hiPSC-derived neurons that can take several weeks, when starting from a NSC line, and longer if the hiPSC cell needs to be created. However, when using reprogrammed neurons derived from patients with a specific disorder, caution needs to be taken in the interpretation of observed effects given the known epigenetic variability and genetic aberrations that occur during reprogramming (discussed in Section 2.2), with further experimentation likely needed to validate conclusions or the proposed hypotheses of relevant parasite/neuronal interactions.

#### 3.2.4. Provides a Model for the Study of Bradyzoite Replication and Cyst Development

Both of these human neuronal models afford the opportunity to study bradyzoite growth and cyst development in neurons, crucial aspects of the parasites biology in the chronic infection for which there are currently no good in vitro models, and that are poorly understood. For example, it has been thought that bradyzoites initially proliferate within cysts, but then become non-proliferative and remain dormant within cysts throughout the chronic infection, but more recent studies indicate that bradyzoite replication and cyst growth actively occur throughout chronic infection, indicating there are dynamic processes that occur in the chronic phase of the infection that are not understood [2,70,85,89,90,91]. The development of an in vitro system that generates cysts and allows cyst maturation to occur, would allow these dynamic aspects of bradyzoite replication and cyst growth to be studied, information that is currently difficult to obtain using in vivo systems. This could have important implications for our understanding of the chronic infection in the brain and significant impacts on treatment strategies for the chronic phase of infection. Additionally, development of human neuronal/cyst model would allow drugs that are effective against bradyzoites and cysts to be studied, which is of importance as there are currently no drugs that effectively target the bradyzoite stage and cysts.

#### 3.2.5. Neuronal-Astrocyte Co-Cultures

In addition to neurons, astrocytes can also be derived from hiPSC-derived direct differentiation method from the NSCs via use of the transcription and growth factors, activin and hergulin. Thus, neuronal-astrocyte co-cultures can be created, which would allow other questions to be addressed. For example, it has been shown that synapse development is promoted when neurons are cultured with astrocytes, and thus neuronal-astrocyte co-cultures could be used to study effects of parasite infection on the synapse [54]. Additionally, neuron-astrocyte interactions have been shown to be important in the impact of the parasite on neurotransmission, thus neuronal-astrocyte co-culture would facilitate the investigation of the effects of infected neurons on neurotransmission, making this is a very attractive aspect of this model [92]. Additionally, astrocytes are known to function as important immune effector cells in the brain, and have been shown to be essential for controlling the chronic phase of *T. gondii* in the brain, although the mechanistic basis is not fully understood [93,94,95]. Astrocyte-neuronal models would allow the interaction of astrocytes and infected neurons to be more broadly explored, and thus may contribute to our understanding of the intracerebral immune responses controlling the chronic infection.

## 4. Conclusions

A better understanding of the effects of chronic Cerebral Toxoplasmosis in the brain, and the underlying mechanisms affecting human behavior and neurological functions has been hindered by the lack of suitable human neuronal models. Murine models of chronic toxoplasmosis, or in vitro models in non-human neuronal cells do not necessary recapitulate the human phenotype of neurological disorders, due to the inherent structural, developmental, and behavioral differences between mice and human nervous systems. The advent of hiPSC technology has been especially useful for the study of neurobiological disorders, where research has similarly been complicated by lack of access of neuronal tissues and the complex nature of many neurological disorders. These cellular reprogramming-based human neuronal models provide similar opportunities to address pathogenesis, disease mechanisms, and drug screening for Cerebral Toxoplasmosis. Additionally, the use of patient specific neurons in these models allows the effect of the parasite on neurons to be studied in a disease-specific context. Use of these human neuronal models has the potential to enhance our understanding of chronic Cerebral Toxoplasmosis and its effects on various neurological disorders, as well as possibly leading to more effective treatments.

## Figures and Tables

**Table 1:** Advantages of reprogrammed neurons with potential applications for research on host/parasite interactions in neurons and biology of parasite in the brain.

Advantage	Neuron Method	Example of Possible Application
Unlimited supply of neurons	hiPSC-neurons or iNs	♦ Dissection of tachyzoite/neuron interactions ♦ Determination of the effects of interferon–gamma (IFN-γ) on tachyzoite replication, stage conversion, etc.
Generation of mature functional neurons	hiPSC-neurons or iNs	♦ Probe tachyzoite and bradyzoite effects on neurotransmission and action potentials
Creation of disease specific neurons	hiPSC-neurons or iNs ^2^	♦ Study of parasite effects on neurons in a disease specific context (i.e., Schizophrenia specific neurons, Parkinson’s specific neurons, etc.)
Generation of in vitro model of bradyzoite growth/cyst development	hiPSC-neurons ^1^ or iNs	♦ Dissectin of bradyzoite/neuron interactions ♦ Study dynamics of bradyzoite replication and cyst growth ♦ Drug discovery targeting bradyzoites and cysts
Creation of Neuron-Astrocyte Co-cultures	hiPSC-neurons ^1^ or iNs	♦ Study the effects of tachyzoite and bradzyoite stages on the synapse/neurotransmission ♦ Investigation of interactions between infected neurons and astrocytes

^1^ hiPSC-derived neurons may be better for this purpose; ^2^ iNs may be better for this purpose.
